# Glucocorticoid‐induced hyperglycaemia in hospitalised adults: A matched cohort study (2013–2023)

**DOI:** 10.1111/dom.16378

**Published:** 2025-04-02

**Authors:** Rajna Golubic, Hudson Mumbole, Ruth L. Coleman, Rustam Rea, Rohini Mathur, Rishi Caleyachetty, Amanda I. Adler

**Affiliations:** ^1^ Diabetes Trials Unit, Radcliffe Department of Medicine University of Oxford Oxford UK; ^2^ Oxford Centre for Diabetes, Endocrinology and Metabolism Oxford University Hospitals NHS Foundation Trust Oxford UK; ^3^ Wolfson Institute of Population Health Queen Mary University of London London UK; ^4^ Warwick Medical School University of Warwick Coventry UK

**Keywords:** cohort study, database research, drug mechanism, insulin therapy, pharmaco‐epidemiology, real‐world evidence

## Abstract

**Aims:**

To compare the risk of new‐onset hyperglycaemia between inpatients treated versus non‐treated with systemic glucocorticoids and identify factors associated with glucocorticoid‐induced hyperglycaemia (GIH).

**Materials and methods:**

We conducted a cohort study using electronic healthcare records of adults admitted to the Oxford University Hospitals between 2013 and 2023. We excluded patients with diabetes or prescribed systemic glucocorticoids before admission. The outcome was new‐onset hyperglycaemia defined as a new glucose‐lowering therapy, coded diagnosis of diabetes or random blood glucose ≥11.1 mmol/L. We used Poisson regression to estimate the incidence rate ratio (IRR) of new‐onset hyperglycaemia during periods of exposure versus non‐exposure to systemic glucocorticoids, adjusting for confounders. We used Poisson regression models to identify potential risk factors for GIH.

**Results:**

Of 451 606 included patients, 17 258 (3.8%) received systemic glucocorticoids during admission. Totally 316 (1.8%) of patients exposed to systemic glucocorticoids developed new‐onset hyperglycaemia versus 3430 (0.8%) non‐exposed to systemic glucocorticoids. The multivariable‐adjusted IRR (95% CI) for new‐onset hyperglycaemia among exposed versus non‐exposed was 2.15 (1.18–3.12). Covariates associated with GIH were: age (relative risk, 95% CI) 1.02 (1.01–1.03) per year, ethnicity (1.72 [1.04–2.86] Asian vs. White, 1.26 [1.05–2.70] other vs. White), weight 1.01 (1.01–1.03) per kg, indication (2.15 [1.21–3.52] autoimmune/inflammatory/infection vs. malignant, 2.11 [1.18–4.20] other vs. malignant) and cumulative glucocorticoid dose (1.23 [1.04–1.42], for 51–205 mg vs. >0–50 mg and 2.53 [1.89–3.40] for > 205 mg vs. >0–50 mg).

**Conclusions:**

Treatment with systemic glucocorticoids versus no glucocorticoid treatment during hospitalisation more than doubles the risk of new‐onset hyperglycaemia. Higher age, weight, cumulative glucocorticoid dose, non‐White ethnicity and autoimmune/inflammatory conditions were independently associated with a higher risk of GIH.

## 1 INTRODUCTION

Glucocorticoids are used commonly for treating autoimmune and inflammatory disorders,^1^ malignancies^2^ and hospitalised patients with COVID‐19^3^ and other diseases that trigger acute respiratory distress syndrome. It has been estimated that ~10% of the hospitalised patients in the United Kingdom (UK) have received systemic glucocorticoids during admission.^4^ Glucocorticoids are associated with adverse metabolic effects including new‐onset diabetes (glucocorticoid‐induced diabetes [GID]).^5^, ^6^ In the UK, ~2% of all newly diagnosed diabetes is related to glucocorticoids^7^ which translates to ~2200 cases/year.^8^

Previous reports suggest that the proportion of people during hospitalisation who develop glucocorticoid‐induced hyperglycaemia (GIH) ranges from 15% to 60%.^2^, ^9^, ^10^, ^11^, ^12^, ^13^, ^14^ One meta‐analysis found that the proportion of GIH was 19% using data from small retrospective studies.^15^ Epidemiologic studies focusing on GIH and its risk factors among inpatients were conducted on small samples of patients with a single category of conditions including inflammatory bowel disease,^14^ respiratory diseases^9^, ^13^ and haematologic malignancies.^2^ Evidence related to incidence from large‐scale rigorously designed studies of inpatients with GIH across a range of indications for glucocorticoids is lacking.

Among inpatients, hyperglycaemia and glucose variability are independently associated with prolonged hospitalisation and mortality, acknowledging that patients with the highest glycaemia are likely to be particularly ill.^16^, ^17^ Heterogeneity in pharmacokinetics between different formulations of glucocorticoids leads to different diurnal glycaemic profiles^18^ characterised by the differences in the onset, peak and resolution of hyperglycaemia. Glycaemic trajectories in patients who develop GIH during hospitalisation, and whether they are associated with acute hyperglycaemic complications, are not well described.

Glucose monitoring and glucose‐lowering therapy in GIH have not been comprehensively studied in hospitalised patients. There is no generally accepted approach to manage GIH and different strategies have been described for different settings.^18^, ^19^ In people without diabetes who are started on systemic glucocorticoids, Joint British Diabetes Societies (JBDS) guidance on managing GIH recommends monitoring capillary blood glucose (CBG) once daily and increasing the frequency to four times daily if CBG > 12 mmol/L. Sulphonylureas are recommended if CBG > 12 mmol/L on 2 occasions in 24 h. However, this guidance reflects an evidence base that relied predominantly on expert opinion rather than empirical research.

In this large‐scale study using electronic health records (EHR) of inpatients, our primary objectives were to: (1) quantify the incidence rate ratio (IRR) of new‐onset hyperglycaemia associated with periods of exposure in patients exposed to systemic glucocorticoids compared with matched periods of non‐exposure in patients non‐exposed to systemic glucocorticoids; (2) compare the length of stay (LOS) between those who developed GIH versus those who did not in patients who received systemic glucocorticoids; (3) identify clinical and demographic risk factors for GIH. In secondary objectives, we focused on patients who developed GIH and described: (1) glycaemic trajectories; (2) the frequency of glycaemic monitoring; (3) the type and duration of glucose‐lowering treatment.

## 2 MATERIALS AND METHODS

### 2.1 Study setting and population

We conducted an observational cohort study using routinely collected data from the EHR of 528 787 adult (age ≥ 18 years) inpatients from the Oxford University Hospitals (OUH) National Health Service (NHS) Foundation Trust admitted between 1 January 2013 and 1 October 2023. Patients were excluded if they had a diagnosis of diabetes or a prescription for systemic glucocorticoids before admission.

**FIGURE 1:**
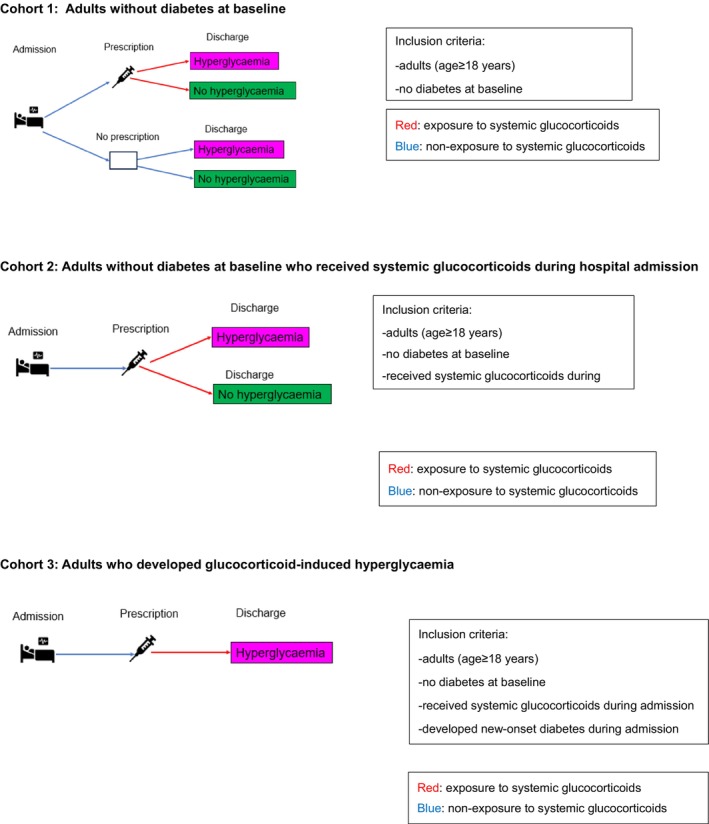
Study cohorts.

We used these data to create 3 cohorts (Figure 1). Cohort 1 included adult inpatients without diabetes at baseline. Cohort 2 included adults without diabetes at baseline who received systemic glucocorticoids during hospitalisation. Cohort 3 included the subset of individuals in Cohort 2 who developed GIH during hospitalisation.

### 2.2 Exposure

Exposure was defined as having a prescription for prednisolone >5 mg/day or equivalent administered systemically (oral, nasogastric, intravenous or intramuscular) during hospitalisation. Table S1 details all glucocorticoids used, systemic routes of administration and equipotent doses.

### 2.3 Outcome

The outcome was new‐onset hyperglycaemia defined as documentation of a new glucose‐lowering therapy, a coded diagnosis of new diabetes (Table S2) or at least 1 random venous blood glucose ≥11.1 mmol/L at any time during hospitalisation.

### 2.4 Statistical analysis

We summarised baseline characteristics using mean (SD) or median (IQR) for continuous variables and *N* (%) for categorical variables.

### 2.5 Primary objectives

#### 2.5.1 IRR (incidence rate ratio)

Using data from adult inpatients without diabetes at baseline (Cohort 1), we fitted Poisson regression models to estimate the IRR (95% CI) of new‐onset hyperglycaemia (the dependent variable) in patients exposed to systemic glucocorticoids versus non‐exposed. Patients were considered non‐exposed until the first prescription for systemic glucocorticoids, after which they were considered exposed. The end date of exposure was defined as the date of the last prescription for glucocorticoids plus 15 days or discharge date, whichever occurred first; the 15‐day period allows for delayed drug‐related effects. People non‐exposed to systemic glucocorticoids were considered unexposed for the entire period of follow‐up. This follow‐up time during periods of non‐exposure started on the calendar day of prescription of the matched individual during periods of exposure, which is referred to as prescription time‐distribution matching.^20^ Time was expressed in days to calculate person‐days.

We also performed one‐to‐one propensity score matching (PSM)^21^ using the greedy nearest neighbour algorithm with replacement as detailed in the Supplementary Methods. Patients were matched for age, sex, weight, ethnicity, indication category for glucocorticoids, LOS and use of medications that increase the risk of diabetes (Table S3). We then fitted Poisson regression adjusting for propensity scores to estimate the IRR (95% CI) of new‐onset hyperglycaemia in patients exposed to systemic glucocorticoids versus non‐exposed.

#### 2.5.2 LOS

Using data from individuals in Cohort 2, we compared median (IQR) LOS for those who developed GIH to those who did not develop GIH using negative binomial regression. We calculated the crude mean ratio and adjusted for the number of comorbidities (1, 2, ≥3).

#### 2.5.3 Risk factors for new‐onset hyperglycaemia in people treated with systemic glucocorticoids

Among adults without diabetes at baseline who received systemic glucocorticoids during hospitalisation (Cohort 2), we used Poisson regression to identify clinical and demographic risk factors for new‐onset hyperglycaemia. We included as potential clinical risk factors the category/tertile of cumulative glucocorticoid dose (mg) (>0–50 [reference], 51–205 and > 205), indication category for glucocorticoids [malignant (reference), autoimmune/inflammatory/infection and other], COVID‐19 status [yes/no (reference)], use of medications associated with an increased risk of diabetes (yes/no (reference); Table S4) and body weight (kg) as a continuous variable. Demographic factors included age at hospitalisation (years), sex [male (reference)/female] and ethnicity [White (reference), African, Asian, other, not stated]. We used backward elimination to select explanatory variables for the final model (*p* = 0.2 threshold) and a likelihood ratio test as a confirmatory method (*p* < 0.2 threshold).

### 2.6 Secondary objectives

#### 2.6.1 Glycaemic trajectories in patients with GIH (Cohort 3)

Using Cohort 3 (individuals who developed GIH during hospitalisation) we used latent class trajectory modelling (LCTM)^22^, ^23^, ^24^ to describe blood glucose trajectories and simplify heterogeneous populations into homogeneous classes, as detailed in the Supplementary Methods.

#### 2.6.2 Frequency of glycaemic monitoring in patients with GIH (Cohort 3)

We used Cohort 3 to calculate the median (IQR) number of times blood glucose was measured daily.

#### 2.6.3 Type and duration of glucose‐lowering treatment in patients who developed GIH

Using Cohort 3, we calculated *N* (%) patients who received different types of glucose‐lowering treatment and the median (IQR) number of days of treatment.

### 2.7 Exploratory analyses

We used multi‐level modelling to account for repeated measures during multiple admissions of the same patient, with clustering by number of admissions, to assess IRR (95% CI) of new‐onset hyperglycaemia in the patients exposed to systemic glucocorticoids versus non‐exposed (Supplementary Methods). We used alternative designs, including self‐controlled case series and case–control design without time‐window bias (Supplementary Methods).

### 2.8 Missing data

Less than 5% of data were missing for ethnicity, so we used complete case analysis^25^ for ethnicity. As data for body weight were missing for >5% and < 50%, we performed multiple imputation for body weight. For indication category that was missing for >50%, we performed mode imputation.

### 2.9 Ethics approval

The Health Research Authority (reference 23/HRA/4185) approved this study. We completed a Data Protection Impact Assessment and obtained approval from the OUH Information Governance team.

## 3 RESULTS

### 3.1 Baseline characteristics

**FIGURE 2:**
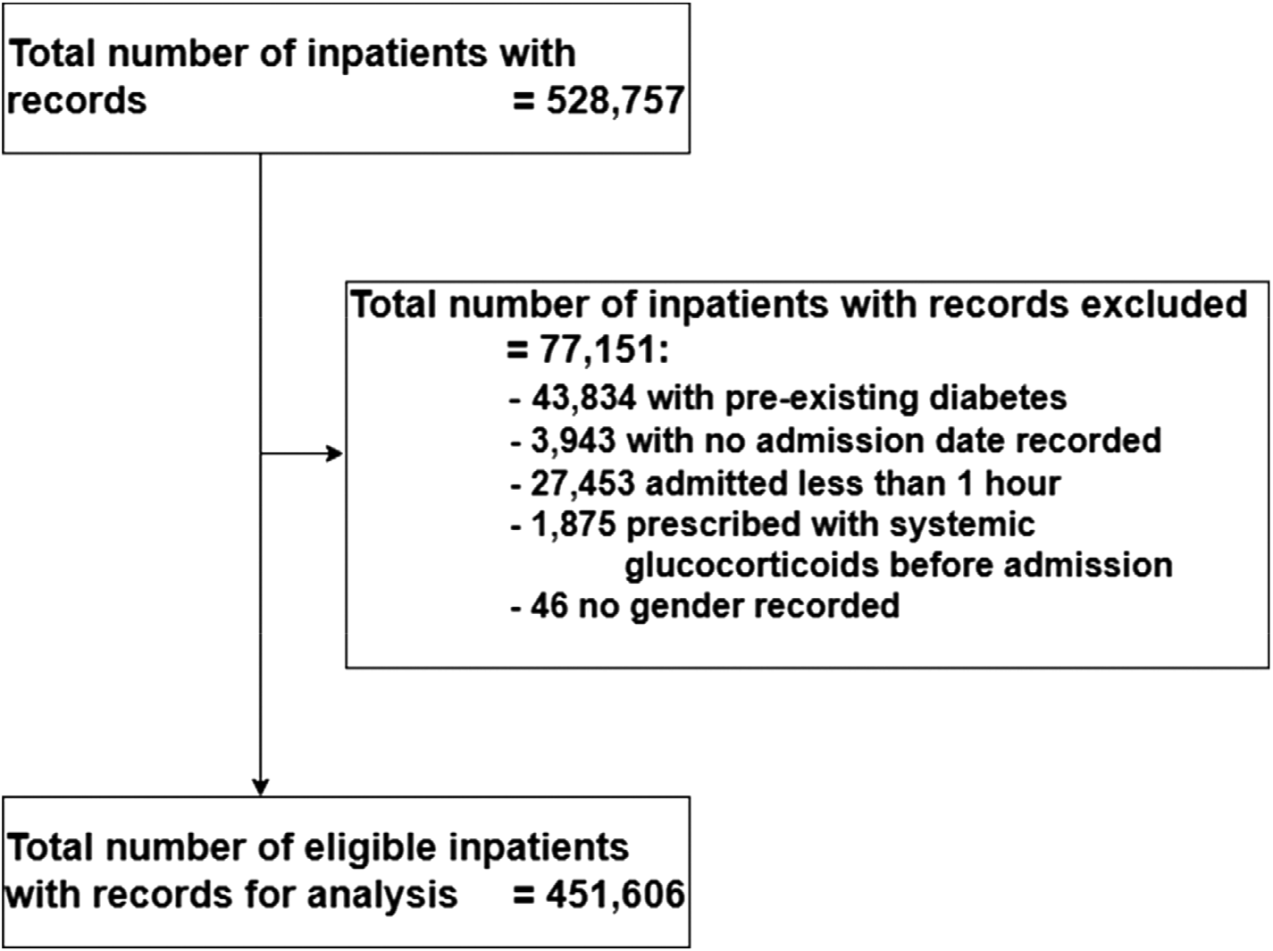
Flow diagram of the study.

Of 528 787 individuals, 451 606 were included in the analysis (Figure 2) with 17 258 (3.8%) exposed and 434 348 non‐exposed to systemic glucocorticoids. Patients were predominantly women of White ethnicity with a median (IQR) age at hospital admission of 52 (34–68) years and the most common indication category for systemic glucocorticoids was autoimmune/inflammatory condition/infection (Table 1).

**TABLE 1:** Baseline characteristics.

	All	Exposed	Non‐exposed	Missing	*p*‐value
*N*	451 606	17 258	434 348		
Age (years) median (IQR)	52 (34–68)	51 (32–68)	52 (34–68)		<0.001^a^
Sex *N* (%)					
Male	201 594 (45)	8163 (47)	193 431 (45)		<0.001^b^
Female	250 012 (55)	9095 (53)	240 917 (55)		
Ethnicity *N* (%)				540 (0.1%)	<0.001^b^
White	310 062 (68.7)	10 709 (62.1)	299 353 (69.0)		
African	4420 (1.0)	216 (1.3)	4204 (1.0)		
Asian	14 863 (3.3)	703 (4.1)	14 160 (3.3)		
Other	9617 (2.1)	403 (2.3)	9214 (2.1)		
Not stated	112 104 (24.9)	5214 (30.2)	106 890 (24.6)		
Body weight (kg), mean (SD)	75 (16)	74 (16)	75 (16)	197 238 (43.7)	0.001^c^
Indication category for systemic glucocorticoids *N* (%)				313 811 (69.5)	0.060^b^
Autoimmune/inflammatory/infection	80 512 (58.4)	5910 (65.3)	74 602 (58.0)		
Malignancies	24 900 (18.1)	1562 (17.3)	23 338 (18.1)		
Other	32 383 (23.5)	1579 (17.4)	30 804 (23.9)		

^a^ Wilcoxon signed‐rank test.

^b^ *χ* ^2^ test.

^c^ Student *t*‐test.

We described glucocorticoid treatment in Table S4. The median (IQR) treatment duration was 3 (1–6) days. The most frequently prescribed glucocorticoid was dexamethasone, *N* = 8764 (50.8% of all glucocorticoid prescriptions). The median (IQR) cumulative dose was 106 (31–320) mg prednisolone‐equivalents (Table S4).

### 3.2 Primary objectives

#### 3.2.1 IRR; Cohort 1

Among the 17 258 exposed patients, 316 (1.8%) developed new‐onset hyperglycaemia over 222 536 person‐days, while among the 434 348 non‐exposed patients, 3430 (0.8%) developed new‐onset hyperglycaemia over 6 760 967 person‐days (Table 2). Crude IRR (95% CI) of new‐onset hyperglycaemia in exposed patients versus non‐exposed to systemic glucocorticoids was 2.80 (2.40–3.05) (Table 2). After adjusting for age and sex, the IRR was 2.60 (2.40–2.90). Multivariable adjustment for age, sex, weight, ethnicity, indication category and use of medications that increase the risk of hyperglycaemia resulted in a further reduction in IRR, but the estimate remained significant: IRR = 2.15 (1.18–3.12). The propensity‐score‐adjusted model yielded IRR = 2.21 (1.27–3.15) (Table 2). Details of sequential adjustment are shown in Table S5.

**TABLE 2:** Incidence rate ratio (95% CI) of new‐onset hyperglycaemia in ever‐exposed patients versus never‐exposed to systemic glucocorticoids based on Poisson regression model.

	*N*	Cases	Person‐days	IR per 100 000 person‐days	Crude IRR (95% CI)	Age‐ and sex‐adjusted IRR (95% CI)	Multivariable‐adjusted IRR (95% CI)	Propensity‐score‐adjusted IRR (95% CI)
Never‐exposed	434 348	3430	6 760 967	50.7	Reference	Reference	Reference	Reference
Ever‐exposed	17 258	316	222 536	142.0	2.80 (2.40–3.05)	2.60 (2.40–2.90)	2.15 (1.18–3.12)	2.21 (1.27–3.15)

*Note*: In the multivariable model, the following variables were adjusted for: age, sex, ethnicity, weight, indication category, LOS and use of medications that increase the risk of diabetes. The [median (IOR)] follow‐up time in days for the never‐exposed and ever‐exposed groups is [3 (1–8)] and [4 (1–9)] respectively.

Abbreviations: IRR, incidence rate ratio; LOS, length of stay.

#### 3.2.2 LOS; Cohort 2

Median (IQR) LOS was 9.0 days (4.5–22.0) in those who developed GIH versus 3.0 days (2.0–8.0) in those who did not. Negative binomial regression model showed that the ratio of estimated mean LOS for those who developed GIH versus those who did not was 2.4 (95%CI 2.1–2.7), *p* < 0.001 and 1.6 (1.1–2.0), *p* = 0.011 after adjusting for the number of comorbidities (Table S6).

We observed an increasing trend in the incidence of GIH with higher LOS (Figure S1).

#### 3.2.3 Risk factors for GIH; Cohort 2

All variables (described in the Methods) except COVID‐19 status and medications which increase the risk of hyperglycaemia were selected to enter the final Poisson regression model (Table 3). Using backward elimination, all selected factors except sex remained significant with RR (95% CI): age (per year) 1.02 (1.01–1.03), ethnicity (1.72 [1.04–2.86] for Asian vs. White, 1.26 [1.05–2.95] for other vs. White), body weight 1.01 (1.01–1.03) per kg, indication category (2.15 [1.21–3.52] for autoimmune/inflammatory/infection vs. malignant, 2.11 [1.18–4.20] for other vs. malignant) and cumulative glucocorticoid dose category (1.23 [1.04–1.42], for 51–205 mg vs. >0–50 mg and 2.53 [1.89–3.40] for > 205 mg vs. >0–50 mg).

**TABLE 3:** Clinical and demographic factors associated with glucocorticoid‐induced hyperglycaemia.

Variable	RR	95% CI
Age (years)	1.02	1.01–1.03
Gender		
Male	ref	1
Female	0.89	0.70–1.12
Weight (kg)	1.01	1.01–1.03
Ethnicity *N* (%)		
White	ref	1
African	1.31	0.48–3.54
Asian	1.72	1.04–2.86
Other	1.26	1.05–2.70
Not stated	1.16	0.91–1.47
Indication category for systemic glucocorticoids (SG)		
Malignancies	ref.	1
Autoimmune/inflammatory/infection	2.15	1.21–3.52
Other	2.11	1.18–4.20
Cumulative dose of systemic glucocorticoids (mg)		
0–50	ref.	1
51–205	1.23	1.04–1.42
>205	2.53	1.89–3.40

### 3.3 Secondary objectives; Cohort 3

#### 3.3.1 Glycaemic trajectories in patients with GIH

Using LCTM we identified a 2‐class model with 2 quadratic trajectories, as illustrated in Figure S1. The model demonstrated satisfactory distribution in each class membership: ‘Class 1’ accounted for 40%, while ‘Class 2’ accounted for 60% (Figure S1) of the total membership. The model's average posterior probability met the recommended minimum of 0.70. The odds of accurate classification of the model, based on the posterior probabilities of class membership, were above 5.0 for both selected classes, indicating a high level of assignment accuracy. The lower BIC of 3017.97 and an entropy of 0.4 indicated lower classification uncertainty compared to other models. Class 1 (*N* = 126, 40%) comprises individuals with slightly lower average glucose concentration compared to Class 2 (*N* = 190, 60%) at glucose measure 1. The average glucose concentration for Class 1 decreased at glucose measure 2, then increased from glucose measure 3–4. On the other hand, the average glucose concentration in Class 2 initially decreased from glucose measure 1–2, and subsequently increased from glucose measure 3, remaining stable until glucose measure 4. Glucose measures are the first four consecutive glucose readings taken after administration of glucocorticoids per individual irrespective of time interval between measurements.

Acute hyperglycaemic complications (diabetic ketoacidosis and hyperosmolar hyperglycaemic state) were recorded in 3 of 316 patients with GIH.

#### 3.3.2 Frequency of glycaemic monitoring in patients with GIH

The median (IQR) number of times blood glucose was monitored daily was 2 (1–4).

#### 3.3.3 Type and duration of glucose‐lowering treatment in patients with GIH

A variety of glucose‐lowering therapies were started in patients with GIH (Table S7). The most commonly prescribed oral glucose‐lowering treatments were metformin, *N* = 103 (33%) and gliclazide, *N* = 87 (28%). The most common insulin prescribed was aspart, *N* = 98 (31%) (Table S7). Median treatment duration across all glucose‐lowering therapies ranged between 1 and 3 days (Table S7).

### 3.4 Exploratory analyses

#### 3.4.1 Multi‐level modelling

To account for multiple admissions in the same patient, we used clustering by admission within a patient from multi‐level modelling and fitted Poisson regression models to assess IRR (95% CI) of new‐onset hyperglycaemia in the patients exposed to systemic glucocorticoids versus non‐exposed (Table S8). The Poisson regression model with clustering within a patient from multi‐level modelling showed a crude IRR of new‐onset hyperglycaemia in exposed versus non‐exposed was 2.4 (1.8–3.0). After adjusting for age, sex, ethnicity, weight, indication category and LOS and use of medications that increase the risk of hyperglycaemia, IRR marginally decreased but remained significant (Table S8).

#### 3.4.2 Self‐controlled case series

In the 316 patients who developed GIH, we defined periods of exposure and non‐exposure to systemic glucocorticoids for each patient and used a self‐controlled case series (SCCS) as detailed in Supplementary Methods. RR (95% CI) for GIH in exposed versus non‐exposed periods was 3.26 (1.90–4.62) (Table S9). Factors significantly associated with GIH were a higher dose and longer duration of glucocorticoid treatment (Table S9).

#### 3.4.3 Case–control design avoiding time‐window bias

After matching for age and sex and fitting a conditional Poisson model (Supplementary Methods), the crude IRR (95% CI) was 2.74 (1.52–3.96) (Table S10). This estimate remained unchanged after adjusting for body weight, ethnicity, indication category and LOS (Table S10).

## 4 DISCUSSION

This study shows that being treated with systemic glucocorticoids during hospitalisation more than doubles the risk of new‐onset hyperglycaemia compared to no glucocorticoid treatment after multivariable adjustment. Among inpatients treated with systemic glucocorticoids, LOS was 1.6 times significantly higher in those who did versus those who did not develop GIH after adjusting for the number of comorbidities. We showed that higher age, Asian or other non‐White ethnicity, higher body weight, autoimmune/inflammatory/infection indication category and higher cumulative dose of glucocorticoids were significant risk factors for GIH.

Previous studies focusing on the risk of new‐onset hyperglycaemia associated with glucocorticoid treatment in hospitalised patients have been conducted on small samples (<200) of patients with a single disease or group of diseases (e.g. inflammatory bowel disease,^14^ chronic obstructive pulmonary disease^13^ or hematologic malignancies^2^) with GIH prevalences between 15 and 60%.^2^, ^13^, ^14^ All included patients were exposed to systemic glucocorticoids during hospitalisation; those with prevalent diabetes were included, and time‐to‐event analyses were not performed. In contrast, our study had a sample size of 451 606 and used time‐to‐event analysis. Therefore, the differences in patients studied (one indication vs multiple indications), inclusion of those with prevalent diabetes and differences in analytical techniques might have explained a lower proportion of those with new‐onset hyperglycaemia in our study. Larger‐scale studies of new‐onset diabetes associated with glucocorticoids have been conducted using data from general practice records.^1^, ^26^, ^27^ A large‐scale observational study published in 2022 using primary care data in England reported that incidence rates of T2D occurring up to 3 years after exposure to systemic glucocorticoids for all indications were 12.5 and 6.7 events per 1000 person‐years, respectively, in those who received at least one dose of glucocorticoids versus those non‐exposed, corresponding to RR = 1.85 (95% CI 1.74–1.97).^27^ This study also showed that male sex, obesity, age 46–55 years, long duration and high dose of glucocorticoid treatment were risk factors for incident T2D.^27^ However, this study included adults in the community rather than hospitalised patients. The periods of exposure and non‐exposure for the same patient were not accounted for, and the study was analysed as a matched case–control study. Therefore, the difference from our estimates might be attributable to our population being more unwell and requiring hospitalisation and to different analytical strategies used. In the studies of patients with asthma or autoimmune diseases using the same primary care database, the risk of developing T2D was 1.7–2.9 times higher in those receiving oral glucocorticoids versus not receiving glucocorticoids.^1^, ^26^

Our study has strengths. Firstly, a large sample size allowed us to generate more statistically robust estimates than previous studies. Secondly, we performed sensitivity analyses using alternative study designs, including case–control study avoiding time‐window bias and self‐controlled case series to remove time‐invariant confounding, and demonstrated that the estimated risk of new‐onset hyperglycaemia associated with glucocorticoid treatment remained significantly elevated.

Several limitations need to be considered when interpreting our findings. Since this is an observational study, causality cannot be inferred. Furthermore, bias and confounding must be considered. Immortal time bias arises in pharmacoepidemiologic studies when the period before the start of exposure to a medication is misclassified as exposed time.^28^ This leads to a biased estimate of the association between the exposure and the outcome. We addressed this by using time‐varying exposure, which allows for a patient to be considered non‐exposed until the first prescription to a systemic glucocorticoid, after which the same patient is considered exposed.^28^ The observed longer LOS in those who did versus those who did not develop GIH among all patients treated with systemic glucocorticoids may partly be related to detection bias because patients with longer LOS are more likely to be sicker than those with shorter LOS and have a greater opportunity to have their blood glucose monitored closely, thereby resulting in a higher probability of diagnosing new‐onset hyperglycaemia. We are also aware that longer LOS itself would be associated with a greater opportunity to have diabetes detected. We adjusted the binomial regression model for the number of comorbidities and still observed a 1.6‐fold significantly longer LOS in those who developed GIH, suggesting the effect is independent of the number of comorbidities. Yet, confounding by indication remains a possibility. This occurs when the indication for glucocorticoids (i.e. an underlying condition) is associated with new‐onset hyperglycaemia. For example, an inflammatory condition per se may increase the risk of new‐onset diabetes or hyperglycaemia.^29^ To mitigate this, we adjusted multivariable models for indication category and did not observe a material change in IRR of new‐onset diabetes in the exposed patients to glucocorticoids versus non‐exposed. Although we adjusted our analyses for potential confounders, there is a possibility of residual confounding by factors that were not measured. It is possible that patients requiring multiple hospital admissions were sicker and more likely to develop GIH than those who had one admission during the observation period. To address this, we used clustering by admission from multi‐level modelling and then recalculated IRR of new‐onset hyperglycaemia in exposed patients versus non‐exposed to systemic glucocorticoids and observed no change in the effect estimate. However, this may not have adequately controlled for this difference in risk.

Since data on glucocorticoid prescriptions, diagnoses of new diabetes or glucose control among people with diabetes after discharge were not available, we cannot make any conclusions about the duration or dose of glucocorticoid treatment or glucose‐lowering therapy, or whether hyperglycaemia developed or resolved after discharge. It is possible that the observed hyperglycaemia might have represented stress hyperglycaemia which resolved after discontinuing glucocorticoid treatment, and glucose‐lowering treatment was required temporarily, and we were not able to assess the proportion of these patients or the effect of this on our estimates. On the other hand, long courses of high‐dose systemic glucocorticoids tend to be tapered down rather than stopped abruptly to minimise the risk of adrenal insufficiency. LOS was relatively short in our cohort, and it is possible that the patients who were discharged on systemic glucocorticoids developed new‐onset hyperglycaemia after discharge. This would imply that the incidence we reported might underestimate the true incidence of GIH.

Considering the nature of EHR, we cannot exclude the possibility of misclassification of new‐onset hyperglycaemia. A study of ~187 000 EHR from inpatients hospitalised in multiple institutions in the United States used neural networks to identify new‐onset hyperglycaemia based on laboratory results, diagnostic codes, medications, demographic parameters and admission information.^30^ A panel of blinded physicians reviewed the cases that were discordant between this method and a standard approach based on diagnoses generated by trained coders.^30^ The study found that 4.3% of the studied population had either inaccurate or missing diagnosis of diabetes.^30^ The extent to which misclassification of new‐onset hyperglycaemia affected our IRR estimates depends on the error in calculating the periods of being exposed or not exposed to systemic glucocorticoids. If the incidence of new‐onset hyperglycaemia was underestimated in exposed but not in non‐exposed, the observed IRR might underestimate the true IRR. If the incidence of new‐onset hyperglycaemia were underestimated only in people who were non‐exposed, the observed IRR would have been overestimated. It is possible that new‐onset hyperglycaemia was underestimated in exposed and non‐exposed to a different extent and therefore the degree to which it biases IRR remains unknown.

The hypothesised pathophysiologic mechanism of developing GIH is reduced insulin sensitivity^31^ and increased gluconeogenesis^32^ (de novo glucose synthesis in the liver) through the effects of glucocorticoids on pancreatic beta cells, liver, muscle and adipose tissue. Thus, it is possible that prediabetes and associated metabolic dysfunction before starting systemic glucocorticoid treatment increased the risk of GIH in this study. However, we did not have data on prediabetes before starting glucocorticoid treatment and did not quantify the association between prediabetes and GIH. Importantly, impairment of beta cell function in healthy individuals and in those with diabetes exposed experimentally to glucocorticoids can cause or aggravate hyperglycaemia.^33^ We could not investigate these mechanisms in this study.

It has been established that a single administration of a high‐dose systemic glucocorticoid can cause hyperglycaemia^18^, ^32^ It is also possible that a single occurrence of hyperglycaemia observed after systemic glucocorticoid treatment might have represented stress hyperglycaemia. Therefore, it was not possible to determine the proportion of new‐onset hyperglycaemia associated with stress versus glucocorticoids. Data on post discharge glucose concentration were not available, so we could not determine if glycaemia had normalised. Additionally, it is probable that individuals with hyperglycaemia were more ill than those without hyperglycaemia. Data on HbA1c were not available, as HbA1c in patients without known diabetes is not monitored in the NHS in the United Kingdom. We did not have data on the severity of illness requiring systemic glucocorticoid treatment and could not analyse how this affects new‐onset hyperglycaemia. In a sensitivity analysis where blood glucose concentration was excluded from the outcome definition, our estimates remained unchanged.

We used PSM which is a statistical technique used in pharmacoepidemiology that approximates randomisation^21^ and aims to balance treatment groups with respect to characteristics associated with the outcome of interest, while a propensity score represents a conditional probability of receiving treatment based on given characteristics.^21^ The exposed and non‐exposed people to systemic glucocorticoids were matched for age, sex, body weight, ethnicity, indication category for glucocorticoids, LOS and use of medications that increase the risk of hyperglycaemia. The propensity‐score‐adjusted IRR (95% CI) of 2.21 (1.27–3.15) is lower than the crude estimate but remained statistically significant, suggesting a clinically substantial 2.2 increased risk of new‐onset hyperglycaemia in patients exposed to systemic glucocorticoids compared with patients non‐exposed.

We planned to assess the factors associated with acute hyperglycaemic complications (diabetic ketoacidosis and hyperosmolar hyperglycaemic state) in patients with GIH. However, only 3 of 316 patients with GIH developed acute hyperglycaemic complications, which did not allow us to fit a regression model.

Using LCTM, we identified 2 distinct glycaemic trajectories during hospitalisation in patients with GIH. The fluctuations in average glucose concentration could be partly explained by the differences in pharmacokinetics between glucocorticoids, the heterogeneity in glucose‐lowering treatment and its timing in relation to carbohydrate intake.

In conclusion, we demonstrated that being treated with systemic glucocorticoids during hospitalisation increases the risk of in‐hospital new‐onset hyperglycaemia by an estimated 2.2 times compared with no treatment with systemic glucocorticoids. Among patients treated with systemic glucocorticoids, those who developed GIH had longer hospital stays than those who did not develop GIH. Risk factors for GIH were higher age, higher body weight, higher doses of systemic glucocorticoids and non‐White ethnicity. Our findings raise awareness among hospital clinicians and patients about the risk of new‐onset hyperglycaemia associated with systemic glucocorticoid therapy. These results can help clinicians treating patients with systemic glucocorticoids to plan care, design local policies and consider monitoring blood glucose before, during and after the treatment course.

## AUTHOR CONTRIBUTIONS

RG and AIA conceptualized the study. HM performed statistical analysis supervised by RLC. RM, RR, and RC provided specialist advice. RG wrote the first draft of the manuscript. All authors interpreted the study data and edited, reviewed and approved the final version of the manuscript. RG is the guarantor of this work and, as such, had full access to all the data in the study and takes responsibility for the integrity of the data and the accuracy of the data analysis.

## FUNDING INFORMATION

This study was funded by The Association of British Clinical Diabetologists (ABCD). The funder was not involved in the work reported in this paper, including study design, data collection, data analyses, preparation of the manuscript or publication decisions.

## CONFLICT OF INTEREST STATEMENT

The authors declare no conflict of interest.

## PEER REVIEW

The peer review history for this article is available at https://www.webofscience.com/api/gateway/wos/peer-review/10.1111/dom.16378.

## Supporting information


**Data S1.** Supporting Information.

## Data Availability

Study data are confidential due to privacy policy.
