# Supplementary material for: Glucocorticoid‐induced hyperglycaemia in hospitalised adults: A matched cohort study (2013–2023)
Source: Diabetes Obes Metab. 2025 Apr 2;27(7):3635–44. doi: 10.1111/dom.16378 (PMC12146452; doi:10.1111/dom.16378)
Supplement: Supplementary file 1 — Data S1. Supporting Information. [file DOM-27-3635-s001.docx]

**Supplementary Methods**

1. Propensity score matching
2. Missing data analysis
3. Latent class trajectory modelling
4. Multi-level modelling
5. Self-controlled case series design
6. Case-control design avoiding time window bias

**Supplementary Tables**

Table S1 Glucocorticoids and their prednisolone equivalent dose

Table S2 Diagnostic codes for new diabetes

Table S3 Medications associated with an increased risk of diabetes

Table S4 Characteristics of systemic glucocorticoid treatment and use of medications which increase the risk of hyprglycaemia

Table S5 Incidence rate ratio (95% CI) of new-onset hyperglycaemia in the exposed patients *vs.* non-exposed to systemic glucocorticoids based on Poisson regression model with details of sequential adjustments

Table S6 Negative binomial regression model to assess the effects of developing glucocorticoid-induced hyperglycaemia *vs*. not developing on the length of stay among those who have received systemic glucocorticoids

Table S7 Type and duration of glucose-lowering treatment in patients with glucocorticoid-induced hyperglycaemia

Table S8 Incidence rate ratio (95%CI) of new-onset hyperglycaemia in exposed vs non-exposed patients to systemic glucocorticoids based on Poisson regression model with clustering by admission within a patient from multilevel-modelling

Table S9 Factors associated with glucocorticoid-induced hyperglycaemia using self-controlled case series design

Table S10 Incidence rate ratio (95%CI) of new-onset hyperglycaemia in exposed patients vs non-exposed to systemic glucocorticoids based on Poisson regression model using case-control design avoiding time-window bias with matching on age and sex

**Supplementary Figures**

Figure S1 Glycaemic trajectories in people with glucocorticoid-induced diabetes

**Other Supplements**

STROBE Checklist

**Supplementary Methods**

**Propensity score matching**

Propensity score is defined as the conditional probability of being exposed to a variable of interest given a set of observed covariates [1]. In this case, the exposure of interest is treatment with corticosteroids. Patients exposed and non-exposed are matched based on estimated propensity scores at a certain time point during admission. Consequently, this balances the distribution of the measured confounding factors defining the propensity scores between the two groups of exposed and non-exposed [2].

To control for potential confounding, we did one-to-one propensity score matching using the greedy nearest neighbour matching algorithm with replacement [3]. We chose this method because of limited number of exposed patients compared to patients non-exposed to systemic glucocorticoids. We estimated the propensity score, or the probability of exposure to systemic glucocorticoids, by using a logistic regression model with age, gender, weight, ethnicity, LOS, indication category for systemic glucocorticoids and use of medications that increase the risk of diabetes as covariates. We evaluated the distribution of covariates between exposed and non-exposed groups by using the standardised mean difference, considering a value less than 0.1 on the absolute scale to indicate well balanced groups [4]. Using Poisson regression adjusting for assigned propensity scores [5,6] we estimated the IRR (95% CI) of new-onset hyperglycaemia among exposed vs non-exposed to systemic glucocorticoids.

**Missing data analysis**

We used complete case analysis if the proportion of missing data was ≤5%. If the proportion of missingness for a variable was 5%-50% [7] we used multiple imputation. We employed multiple imputation [7] to estimate missing values for body weight. We applied multiple imputations with chained equations with generation of 10 data sets to minimize bias because of missing data [7]. The multiple imputation method for handling missing data involves replicating the original dataset multiple times (10 times in this case) and in each replication replacing the missing values with plausible observations drawn from the posterior predictive distribution [8]. This method is typically conducted using the ‘missing at random’ (MAR) assumption [7], which also includes ‘missing completely at random’ (MCAR). MCAR assumes that missingness does not depend on the observed or missing values while MAR assumes missingness is independent of the observed missing values

To test for pattern of missingness, we used Little’s test [9]. Little’s test tests the null hypothesis that the missing data is MCAR. A p-value of less than 0.05 is usually interpreted as being that the missing data is either missing at random or cannot be ignored.

To achieve <50% threshold for the proportion of missing for indication category for glucocorticoids variable we replaced the missing values with the most frequent (mode [10]) indication among inpatient of same age and sex. We then used multiple imputation to impute missing values for indication category for glucocorticoids and body weight. Body weight was used a proxy for body mass index; this is because the pattern of missingness was not at random for body mass index variable.

We performed a sensitivity analysis by excluding the indication category for glucocorticoids variable.

**Latent class trajectory modelling**

We used a latent class trajectory model (LCTM) [11, 12,13] to describe blood glucose trajectories and simplify heterogeneous populations into homogeneous classes. We determined the number of classes by considering [11,12,13]: lowest value of Bayesian information criterion (BIC), average posterior probability of assignment (APPA) >70%, odds of correct classification (OCC) > 5.0, relative entropy >0.5, the proportion of estimated trajectory classes (the smallest group includes ≥5% of individuals), and clinical meaning of trajectories. We used random quadratic proportional model to allow for individual variation within these classes. We built several models to test for different trajectory shapes until the best fitting models were established.

According to Joengbloed *et al.* [14] to perform the LCTM analysis, we followed the following steps:

1. We created a hypothesis of a plausible number of groups based on theory/literature.

2. We refined the model from step 1 to determine:

a) The optimal number of groups, typically testing K=1- 4 groups.

b) The optimal shape of the trajectories, typically testing linear, quadratic, and cubic functions of each trajectory.

3. We then assessed model fit using Bayesian information criteria (BIC) values, average posterior probability of assignments (APPA), and odds of correct classification (OCC).

4. We investigated graphical presentations and assessed for substantive interpretation.

We used the first 4 glucose measures after the diagnosis of GID regardless of time interval in which they were taken to construct the trajectories. To select which functions best fit the data, we used the following criteria for a good model fit [14]:

1. The Average Posterior Probability Assignment (APPA) of each group to belong to a trajectory examines whether individuals are assigned with high probability and the overall average probability of assignment to each group. The criterion for a good model is that the APPA > 70% for each class.

2. Odds of correct classification (OCC) is the proportion of the odds of a correct classification in each group based on the maximum likelihood classification rule and the estimated proportions of class members. The criterion for a good model is that OCC > 5.0 for each class.

3. Entropy is a global measure of classification uncertainty, which takes into account all subsequent probabilities. Entropy takes values from (0, ∞), with higher values indicating greater uncertainty. The criterion of a good model is when the entropy values are closer to 0 (since they correspond to models with lower classification uncertainty).

4. The optimal group is the one that has the lowest BIC value and does not contain groups smaller than 5%

**Multi-level modelling**

We repeated the analyses described for the primary objective 1 using multilevel-modelling based on clustering by admission within a patient as shown in Table S11.

To investigate these relationships at the patient level, we constructed a statistical model step-by-step, initially assuming hospital admissions as a fixed effect without taking individuals clustering within admission into consideration. Then, using hospital admissions as a distinct random effect, we calculated the variation partition coefficient (VPC) to determine what proportion of the variance in the result was explained by variations in patient characteristics and variations in the number of admissions. Using Austin *et al.* [15, 16, 17] technique, we computed the VPC in this adjusted multilevel Poisson regression model. This is the percentage of the (unexplained) variation in the result caused by variation between clusters. Next, we calculated the VPC values for a typical patient in the study population who was at low and high risk of getting new-onset hyperglycaemia.

We then computed hospital admission level adjusted IRR [15] for models shown in table S11 below.

**Self-controlled case series (SCCS) design**

The SCCS method is an epidemiological study design for which individuals act as their own control, i.e. comparisons are made within individuals [18]. Hence, only individuals who have experienced an event (cases) are included which eliminates all time-invariant confounding [18].

In SCCS design, the relative risk during the exposure (risk) and non-exposure (control) periods are estimated [19]. We defined periods of exposure and non-exposure to systemic glucocorticoids. The observation period used in the SCCS design for each patient was firstly divided into two periods: the period when patients were non-exposed to systemic glucocorticoids (non-exposed period) and the total period of systemic glucocorticoid treatment, defined as the date treatment started until the end date of the prescription/discharge.

Next, we separated a period immediately before the start of treatment that is, time before glucocorticoids were prescribed [20]. In total, the exposure to systemic glucocorticoids period was separated into 3 periods: (i) No systemic glucocorticoid treatment (ii) ≤1 day immediately after glucocorticoid treatment, (iii) >1 day with glucocorticoid treatment.

We also ranked drugs according to their degree of anti-inflammatory activity: cortisone, hydrocortisone, prednisolone, methylprednisolone and dexamethasone [21]. To address the differences in potency between glucocorticoids, we standardised the daily doses to prednisolone equivalents using the information published in the British National Formulary [21]. Next, we calculated the initial daily dose and cumulative dose. We divided the initial daily dose into four categories of clinical effect [22], as 0, 5.0-7.4 and ≥7.5 mg equivalent prednisolone dose/day. The cumulative dose was categorised into 3 groups, as 0, ≤50, 51–205 and > 205 mg equivalent prednisolone dose. Incidence rate ratios (IRRs) and 95% confidence intervals (95% CIs) for new-onset hyperglycaemia were estimated using conditional Poisson regression [23] for the total period with glucocorticoid treatment and for the sub-periods, as compared with the unexposed period. IRRs for new-onset hyperglycaemia were also estimated for types of systemic glucocorticoids and initial and cumulative prednisolone equivalent dose shown in table S12.

**Case-control design avoiding time window bias**

The time window bias is the bias resulting from the use of time-windows of different lengths between cases and controls to define time-dependent exposures [24]. To avoid this bias we equated the duration of follow-up time in the cases and controls [24].

Subsequently, patients who developed new-onset hyperglycaemia (cases) in exposed and non-exposed groups were matched with those that did not develop new-onset hyperglycaemia (controls) in their respective groups on age and sex in the ratio 1:6 [25]. Then incidence rate ratios (IRR) and 95% confidence intervals (95% CI) for new-onset hyperglycaemia among exposed *vs* non-exposed to systemic glucocorticoids adjusted for weight (kg), length of stay (days), ethnicity, indication category for systemic glucocorticoids and use of medications that increase the risk of diabetes as covariates were estimated using conditional Poisson regression [23] as shown in table S13.

**Supplementary Tables**

**Table S1 Glucocorticoids and their prednisolone equivalent dose**

| **Name** | **Dose (mg)** | **Systemic routes of administration to be included in the analysis** |
| --- | --- | --- |
| Budesonide | 0.55 | PO |
| Cortisone | 25 | PO, IV, IM |
| Deflazacort | 6 | PO |
| Dexamethasone | 0.8 | PO, IV, IM |
| Hydrocortisone | 20 | PO, IV, IM |
| Methylprednisolone | 4 | PO, IV |
| Plenadren | 20 | PO, IV |
| Prednisolone | 5 | PO |
| Prednisone | 5 | PO |
| Triamcinolone | 4 | IM |

The conversions are based on the British National Formulary (National Institute for Health and Care Excellence. British National Formulary. London: NICE, 2022.).

**Table S2 Diagnostic codes for new diabetes**

| ICD code | Diagnosis |
| --- | --- |
| E10 | Type 1 diabetes |
| E11 | Type 2 diabetes |
| E13 | Other specified diabetes |
| E14 | Unspecified diabetes |
| O24 | Diabetes in pregnancy |

**Table S3 Medications associated with an increased risk of diabetes**

| **Medication class** | **Medication name** |
| --- | --- |
| Selective serotonin reuptake inhibitors | citalopram, escitalopram, fluoxetine, paroxetine, sertraline, dapoxetine, fluvoxamine, vortioxetine |
| Antipsychotics | amisulpiride, aripriprazole, clozapine, lurasidone, olanzapine, paliperidone, quetiapine, risperidone; chlorpromazine, flupentixol, haloperidol, levopromazine, periciazine, perphenazine, pimozide, chlorperazine, promazine, suppiride, trifluperazine, zuclopenthixol |
| Antiretrovirals | abacavir, didanosine, lamivudine, stavudine, tenofovir, zidovudine; delavirdine, efavirenz, nevirapine, rilpivirine; atazanavir, darunavir, indinavir, ritonavir, cobicistat |

NB Other medications used less frequently that have also been reported to increase the risk of diabetes were not investigated, e.g. somatostatin analogues and tyrosine kinase inhibitors.

**Table S4 Characteristics of systemic glucocorticoid treatment and use of medications which increase the risk of diabetes**

|  | **All** | **Developed new-onset** hyperglycaemia | **Did not develop new-onset** hyperglycaemia |
| --- | --- | --- | --- |
| N | 17,258 | 316 | 16,942 |
| Length of stay, median (IQR) | 3 (2-8) | 9 (5-22) | 3 (2-8) |
| Duration of systemic glucocorticoids treatment days, median (IQR) | 3 (1-6) | 3 (1-7) | 3 (1-6) |
| Type of systemic glucocorticoids N (%):  Budesonide  Cortisone  Dexamethasone  Hydrocortisone  Methylprednisolone  Prednisolone  Prednisone | 74 (0.4)  929 (5.4)  8,764 (50.8)  2,055 (11.9)  370 (2.1)  5,052 (29.3)  14 (0.1) | 0 (0.0)  12 (3.8)  105 (33.2)  63 (19.9)  10 (3.2)  126 (39.9)  0 (0.0) | 74 (0.4)  917 (5.4)  8,659 (51.1)  1,992 (11.8)  360 (2.1)  4,926 (29.1)  14 (0.1) |
| Systemic glucocorticoid dose per day (mg), median (IQR) | 50 (30-100) | 50 (38-100) | 50 (30-100) |
| Systemic glucocorticoid daily dose (mg/day), N (%):  >0-4.9  5.0-7.4  ≥7.5 | -  141 (0.8)  17,117 (99.2) | -  3 (1.0)  313 (99.0) | -  138 (0.8)  16,804 (99.2) |
| Systemic glucocorticoid cumulative dose (mg), median (IQR) | 106 (31-320) | 309 (91-808) | 104 (31-311) |
| Systemic glucocorticoid cumulative dose (mg), N (%):  0 - 50  51-205  > 205 | 5,753 (33.4)  5,753 (33.4)  5,752 (33.2) | 61 (19.3)  78 (24.7)  177 (56.0) | 5,692 (33.6)  5,675 (33.5)  5,575 (32.9) |
| Prescription to non-systemic glucocorticoids N (%):  No  Yes | 14,080 (82)  3,178 (18) | 148 (47)  168 (53) | 13,932 (82)  3,010 (18) |
| Prescription to medications associated with increased risk of diabetes, N (%):  No  Yes | 14,035 (81)  3,223 (19) | 198 (63)  118 (37) | 13,837 (82)  3,105 (18) |

Note: A full list of medications associated with an increased risk of hyperglycaemia is provided in Table S3. Length of stay [median (IQR)] in the unexposed group comparing those who developed new-onset hyperglycaemia and those who did not were [5 (2-13)] and [3 (2-9)], respectively.

**Table S5 Incidence rate ratio (95% CI) of new-onset hyperglycaemia in exposed patients *vs.* non-exposed to systemic glucocorticoids based on Poisson regression model with details of sequential adjustments**

|  | N | Cases | Person-years | IR per 100 person-years | Crude IRR (95% CI) | IRR (95% CI) adjusted for age and sex | IRR (95% CI) adjusted for age, sex, ethnicity and weight | IRR (95% CI) adjusted for age, sex, ethnicity, weight and indication category | IRR (95% CI) adjusted for age, sex, ethnicity, weight, indication category and LOS | IRR (95% CI) adjusted for age, sex, ethnicity, weight, indication category, LOS and use of medications that increase the risk of diabetes | IRR (95% CI) adjusted for propensity score |
| --- | --- | --- | --- | --- | --- | --- | --- | --- | --- | --- | --- |
| Non- exposed | 434,348 | 3,430 | 18,510.52 | 18.53 | Reference | Reference | Reference | Reference | Reference | Reference | Reference |
| Exposed | 17,258 | 316 | 609.27 | 51.87 | 2.80  (2.40-3.05) | 2.60  (2.40-2.90) | 2.36  (1.38-3.34) | 2.38  (1.17-3.59) | 2.29  (1.31-3.27) | 2.15  (1.18-3.12) | 2.11 (1.31-2.91), 0.019 |

Abbreviations: IRR- incidence rate ratio; LOS- length of stay

**Table S6 Negative binomial regression model to assess the effects of developing glucocorticoid-induced hyperglycaemia (N=316) *vs* not developing (N=16,942) on the length of stay among those who have received systemic glucocorticoids**

| **Level of adjustment** | **Mean ratio** | **95% CI** | **p-value** |
| --- | --- | --- | --- |
| Crude | 2.4 | 2.1-2.7 | <0.001 |
| Adjusted for the number of comorbidities | 1.6 | 1.1-2.0 | 0.011 |

Note: The number of co-morbidities was used as a categorical variable: 1, 2, ≥3

**Table S7 Type and duration of glucose-lowering treatment in patients with glucocorticoid-induced hyperglycaemia (N=316)**

| **Type of glucose lowering treatment** | **N (%)** | **Duration in days, median (IQR)** |
| --- | --- | --- |
| Oral therapies | | |
| Gliclazide | 87 (28) | 1 (1-3) |
| Dapagliflozin | 35 (11) | 2 (2-3) |
| Metformin | 103 (33) | 1 (1-4) |
| Empagliflozin | 31 (10) | 2 (2-4) |
| Canagliflozin | 26 (9) | 2 (1-4) |
| Linagliptin | 30 (9) | 1 (1-3) |
| Insulins | | |
| Quick-acting insulins | | |
| Aspart | 98 (31) | 2 (1-3) |
| Human insulin | 35 (11) | 3 (2-6) |
| Intermediate-acting insulin | | |
| Isophane | 24 (7) | 2 (1-4) |
| Long-acting insulins | | |
| Detemir | 60 (19) | 2 (2-3) |
| Glargine | 69 (22) | 3 (1-5) |

Note: In addition to diabetes, SGLT2 inhibitors (dapagliflozin, empagliflozin and canagliflozin) can be used for heart failure and chronic kidney disease. Among patients with dapagliflozin prescription, 3 had a diagnosis code for heart failure and 0 had a code for chronic kidney disease. Among patients prescribed empagliflozin, 2 had a diagnosis code for heart failure and 1 had a code for chronic kidney disease. Among patients prescribed canagliflozin, 3 had a diagnosis code for heart failure and 1 had a code for chronic kidney disease.

**Table S8 Incidence rate ratio (95%CI) of new-onset hyperglycaemia in exposed *vs* non-exposed patients to systemic glucocorticoids based on Poisson regression model with clustering by admission within a patient from multilevel-modelling**

| **Variable** | **Number of admissions** | | | **Crude IRR** | **Age- and sex-adjusted IRR (95% CI)** | **Multivariable-adjusted**  **IRR (95% CI)** |
| --- | --- | --- | --- | --- | --- | --- |
|  | **1 (N=210,453)** | **2-3 (N=88,336)** | **≥4 (N=152,817)** |  |  |  |
| Exposure to systemic glucocorticoids:  No  Yes | 201,615 (95.8%)  8,838 (4.2%) | 85,298 (96.6%)  3,038 (3.4%) | 147,435 (96.5%)  5,382 (3.5%) | Reference  2.41 (1.81-3.01) | Reference  2.40 (2.30-2.51) | Reference  2.28 (1.78-2.78) |

Abbreviation: IRR- incidence rate ratio

Multivariable model was adjusted for age, sex, ethnicity, weight, indication category, length of stay and use of medications that increase the risk of diabetes

**Table S9 Factors associated with glucocorticoid-induced hyperglycaemia using self-controlled case series design** **(N=316)**

| **Variable** | **RR** | **95% CI** |
| --- | --- | --- |
| No use of systemic glucocorticoids (unexposed period)  Use of systemic glucocorticoids (exposed period) | Reference  3.26 | 1.90-4.62 |
| Duration of treatment:  No systemic glucocorticoid  ≤1 day  >1 day | Reference  1.30  1.61 | -  1.13-1.47  1.01-2.21 |
| Type of systemic glucocorticoid:  No systemic glucocorticoids  Cortisone  Hydrocortisone  Prednisolone  Methylprednisolone  Dexamethasone | Reference  2.07  1.35  1.22  1.13  1.31 | -  1.40-2.74  1.21-1.49  1.14-1.30  1.07-1.19  1.17-1.45 |
| Dose of systemic glucocorticoid per day (mg/day):  0  5.0-7.4  ≥7.5 | Reference  1.57  1.61 | -  1.23-1.91  1.17-2.05 |
| Cumulative dose of systemic glucocorticoid (mg):  0  ≤ 50  51–205  > 205 | Reference  1.04  1.91  2.21 | -  0.71-1.37  1.08-2.74  1.23-3.19 |

Note: In self-control case series design periods of exposure and non-exposure are analysed in the same person who developed glucocorticoid-induced diabetes.

**Table S10 Incidence rate ratio (95%CI) of new-onset hyperglycaemia in patients exposed *vs* non-exposed to systemic glucocorticoids based on Poisson regression model using case-control design avoiding time-window bias with matching on age and sex**

| Variable | N | Crude IRR (95% CI) | IRR (95% CI) adjusted for ethnicity and weight | IRR (95% CI) adjusted for ethnicity, body weight and indication category | IRR (95% CI) adjusted for ethnicity, body weight, indication category and LOS | IRR (95% CI) adjusted for ethnicity, body weight, indication category, LOS and use of medications that increase the risk of diabetes |
| --- | --- | --- | --- | --- | --- | --- |
| Exposed to systemic glucocorticoids:  No  Yes | 434,348  17,258 | Reference  2.74 (1.52-3.96) | Reference  2.71  (1.63-3.79) | Reference  2.68  (1.81-3.55) | Reference  2.72  (1.72-3.72) | Reference  2.60  (1.32-3.88) |

Note: All IRR (95% CI) were matched on age and sex.

**Figure S1 Glycaemic trajectories in people with glucocorticoid-induced diabetes**


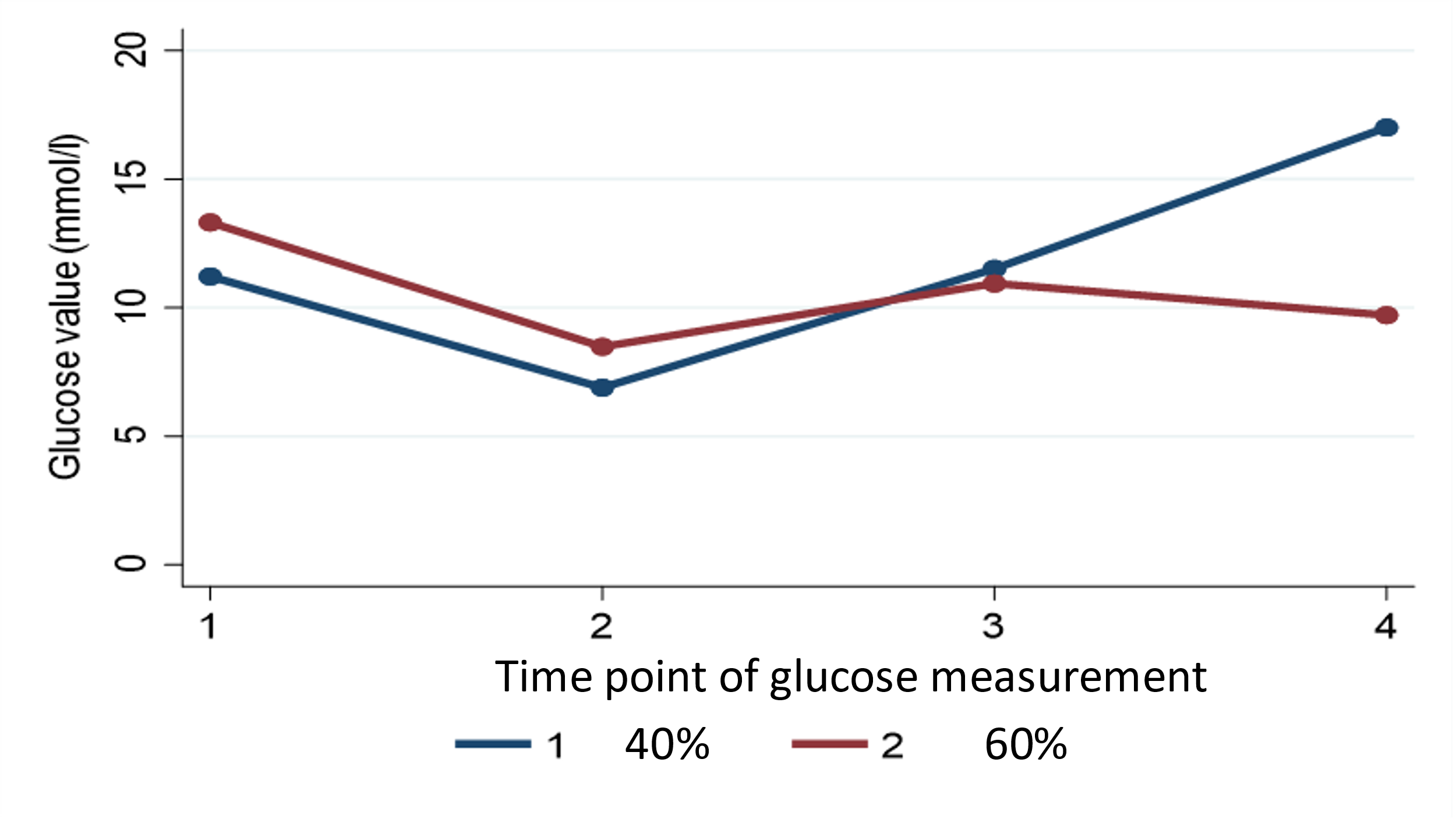


Note: The blue line (legend 1) represents trajectory for 40% of members belonging to class 1 while the red line (legend 2) represents trajectory for 60% of members in class 2 using the criteria in table S3a. Time points represent the order in which blood samples were taken rather than specific time estimates. We were not able to specify the exact timings as these differ materially between the patients.

**Table S3a Trajectory model selection criteria**

| Classes | BIC | Entropy | APPA | OCC | Class<5% |
| --- | --- | --- | --- | --- | --- |
| 1 | -3009.34 | - |  |  |  |
| 2 | -3017.97 | 0.40 | 97% | 12.0 | 0 |
| 3 | -3026.60 | 0.51 | 96% | 11.6 | 1 |
| 4 | -3016.62 | 0.69 | 83% | 10.8 | 1 |

**REFERENCES**

1. Abadie A, Imbens GW. Matching on the estimated propensity score. Econometrica 2016;84:781–807.

2. Shinozaki T, Nojima M. Misuse of regression adjustment for additional confounders following insufficient propensity score balancing. Epidemiology 2019;30:541–8.

3. Rassen JA, Shelat AA, Myers J, Glynn RJ, Rothman KJ, Schneeweiss S (2012). One-to-many propensity score matching in cohort studies. Pharmacoepidemiol Drug Saf 2012;21(Suppl 2):69-80. doi:10.1002/pds.3263

4.Burden A, Roche N, Miglio C, Hillyer EV, Postma DS, Herings RM, Overbeek JA, Khalid JM, van Eickels D, Price DB. An evaluation of exact matching and propensity score methods as applied in a comparative effectiveness study of inhaled corticosteroids in asthma. Pragmat Obs Res. 2017 Mar 22;8:15-30. doi: 10.2147/POR.S122563. PMID: 28356782; PMCID: PMC5367458.

5. Austin PC. An Introduction to Propensity Score Methods for Reducing the Effects of Confounding in Observational Studies. Multivariate Behav Res. 2011 May;46(3):399-424. doi: 10.1080/00273171.2011.568786. Epub 2011 Jun 8. PMID: 21818162; PMCID: PMC3144483.

6. Nicolaides NC, Pavlaki AN, Maria Alexandra MA, et al. Glucocorticoid Therapy and Adrenal Suppression. In: Feingold KR, Anawalt B, Blackman MR, et al., editors. Endotext [Internet]. South Dartmouth (MA): MDText.com, Inc.; 2000.

7. Little RJA, Rubin DB. Statistical Analysis with Missing Data, 2nd edn. United States of America: Wiley; 2002. Online ISBN:9781119013563.

8. Rubin DB. Multiple Imputation for Nonresponse in Surveys. United States of America: Wiley; 1987, p. 258.

9. Little, R. J. A. (1988). A test of missing completely at random for multivariate data with missing values. Journal of the American Statistical Association, 83(404), 1198–1202.

10. Tra My Pham, Nikolaos Pandis, Ian R White. Missing data: Issues, concepts, methods,

Seminars in Orthodontics, Volume 30, Issue 1, 2024, Pages 37-44,ISSN 1073-8746.

11. Liu Q, You N, Pan H, Shen Y, Lu P, Wang J, et al. Glycemic Trajectories and Treatment Outcomes of Patients with Newly Diagnosed Tuberculosis: A Prospective Study in Eastern China. Am J Respir Crit Care Med. 2021;204(3):347-56.

12. Herle M, Micali N, Abdulkadir M, Loos R, Bryant-Waugh R, Hubel C, et al. Identifying typical trajectories in longitudinal data: modelling strategies and interpretations. Eur J Epidemiol. 2020;35(3):205-22.

13. Hulman A, Witte DR, Vistisen D, Balkau B, Dekker JM, Herder C, et al. Pathophysiological Characteristics Underlying Different Glucose Response Curves: A Latent Class Trajectory Analysis From the Prospective EGIRRISC Study. Diabetes Care. 2018;41(8):1740-8.

14. Jongbloed, J. (2021). Group-based trajectory modeling. Complex Trajectories Methodological Group (MGroup). Bourgogne: IREDU, Universitéde Bourgogne

15. Quinn KL, Abdel-Qadir H, Barrett K, Bartsch E, Beaman A, Biering-Sørensen T, Colacci M, Cressman A, Detsky A, Gosset A, Lassen MH, Kandel C, Khaykin Y, Lapointe-Shaw L, Lovblom E, MacFadden DR, Perkins B, Rothman KJ, Skaarup KG, Stall N, Tang T, Yarnell C, Zipursky J, Warkentin MT, Fralick M; COVID-ACE Group. Variation in the risk of death due to COVID-19: An international multicenter cohort study of hospitalized adults. J Hosp Med. 2022 Oct;17(10):793-802. doi: 10.1002/jhm.12946. Epub 2022 Aug 30. PMID: 36040111; PMCID: PMC9539016.

16. Gelman A, and Hill J. Multilevel structures. In: Data Analysis Using Regression and Multilevel/Hierarchical Models. New York City: Cambridge University Press, 2007:chapter11.

17. Kyle M. Kepreos (2015). Multilevel Poisson sample selection models and alternative methods for estimating hospital effects on long-term outcomes. Health Services and Outcomes

18. Petersen I, Douglas I, Whitaker H. Self controlled case series methods: an alternative to standard epidemiological study designs BMJ 2016; 354 :i4515 doi:10.1136/bmj.i4515

19. Takeuchi Y, Shinozaki T, Matsuyama Y. A comparison of estimators from self-controlled case series, case-crossover design, and sequence symmetry analysis for pharmacoepidemiological studies. BMC Med Res Methodol. 2018 Jan 8;18(1):4. doi: 10.1186/s12874-017-0457-7. PMID: 29310575; PMCID: PMC5759844.

20. Meikle AW, Tyler FH. Potency and duration of action of glucocorticoids. Effects of hydrocortisone, prednisone and dexamethasone on human pituitary-adrenal function. Am J Med. 1977;63:200–7.

21. https://bnf.nice.org.uk/treatment-summary/glucocorticoid-therapy.html (accessed 30 May 2024).

22. Hawken, S., Potter, B.K., Little, J. et al. The use of relative incidence ratios in self-controlled case series studies: an overview. BMC Med Res Methodol 16, 126 (2016).

23. Suissa, Samya,b; Dell'Aniello, Sophiea; Vahey, Saraha; Renoux, Christela. Time-window Bias in Case-control Studies: Statins and Lung Cancer. Epidemiology 22(2):p 228-231, March 2011. | DOI: 10.1097/EDE.0b013e3182093a0f

24. Suissa S, Dell'Aniello S, Vahey S, Renoux C. Time-window bias in case-control studies: statins and lung cancer. Epidemiology. 2011;22 (2):228-231.

25. Masao Iwagami,Tomohiro Shinozaki (2022). Introduction to Matching in Case-Control
and Cohort Studies.
